# Human medical documentation significantly outperforms ChatGPT‐4o in critical clinical dimensions: A blinded comparative assessment in paediatric orthopaedics

**DOI:** 10.1002/ksa.70357

**Published:** 2026-02-28

**Authors:** Carlo Camathias, Kata Papp, Patrick Betschart, Berhard Speth, Victor Valderrabano, Elias Ammann

**Affiliations:** ^1^ Praxis Momentum St. Gallen Switzerland; ^2^ Medical Faculty University of Basel Basel Switzerland; ^3^ Orthopaedic Departement Schulthess Clinic Zürich Switzerland; ^4^ Urosteph St. Gallen Switzerland; ^5^ Kinder‐ und Jugendorthopädie Aarau Switzerland; ^6^ Swiss Ortho Center Basel Switzerland; ^7^ Orthopaedic Departement Kantonsspital Baselland Standort Bruderholz Bruderholz Switzerland

**Keywords:** artificial intelligence in healthcare, ChatGPT‐4o, clinical decision support, documentation accuracy, large language model, medical documentation quality, paediatric orthopaedics

## Abstract

**Purpose:**

This study evaluated the quality of ChatGPT‐generated medical history summaries compared to human‐created documentation in a paediatric orthopaedic practice setting.

**Methods:**

A prospective, randomised, blinded comparative study was conducted involving 20 consecutive paediatric patients (mean age 14.2 ± 2.3 years; 11 males, 9 females) presenting with knee problems. Audio recordings of medical consultations were transcribed and processed by ChatGPT‐4o (OpenAI) using standardised prompts. Three independent orthopaedic specialists evaluated both human‐generated and AI‐generated summaries using eight quality criteria: temporal consistency, spatial consistency, accident description, symptom accuracy, symptom specificity, previous interventions, writing style and overall impression. Each criterion was scored on a 6‐point Likert scale.

**Results:**

Human‐created summaries received significantly higher overall ratings (5.2 ± 0.8) compared to ChatGPT‐generated summaries (4.5 ± 0.8, *p* < 0.001, Cohen's *d* = 0.80). After Bonferroni correction for multiple comparisons, statistically significant differences favouring human documentation were confirmed in four of eight criteria: temporal consistency (*p* < 0.001), spatial consistency (*p* < 0.001), accident description (*p* < 0.001) and overall impression (*p* < 0.001). No significant differences were observed for writing style and documentation of previous interventions. Inter‐rater reliability was moderate (ICC = 0.64). ChatGPT demonstrated frequent temporal inconsistencies (14 of 60 evaluations, 23%) and omission of relevant accident details (21 of 60 evaluations, 35%).

**Conclusion:**

While AI‐generated summaries showed acceptable stylistic quality, human documentation significantly outperformed ChatGPT in critical clinical dimensions, including temporal consistency and accuracy of complex orthopaedic presentations. Current large language models are not ready to replace human medical documentation in paediatric orthopaedic practice without careful oversight. The findings support the implementation of hybrid workflows where AI assists but does not replace human clinical judgement.

**Level of Evidence:**

Level I.

**Abbreviations**
AI: artificial intelligence
CI: confidence interval
EEG: electroencephalography
EKOS: Ethikkommission Ostschweiz (Ethics Committee of Eastern Switzerland)
HIPAA: Health Insurance Portability and Accountability Act
ICC: intraclass correlation coefficient
LLM(s): large language model(s)
SD: standard deviation

## INTRODUCTION

Medical documentation represents a significant administrative burden for orthopaedic surgeons, with physicians spending approximately 24% of working hours on administrative duties [13, 17]. Large language models (LLMs) such as ChatGPT have emerged as potential solutions for reducing this burden [1].

Recent evaluations have yielded mixed results [6]. Williams et al. [18] reported that GPT‐4‐generated emergency department summaries contained hallucinations in 42% and clinically relevant omissions in 47% of cases [14]. Butler et al. [2] demonstrated that AI can improve readability of radiology reports, while Çakmur et al. [3] found that Gemini 1.5 Flash provides the most reliable content for patient education on meniscal tears.

Paediatric orthopaedics presents unique challenges: histories involve multiple informants, developmental considerations and growth‐related factors. A fundamental limitation of transcript‐based AI systems is their inability to capture nonverbal communication signals that experienced surgeons routinely incorporate into assessments [16].

Despite growing interest in AI‐supported documentation, systematic quality comparisons are lacking in orthopaedic literature. This gap is clinically relevant because documentation errors may affect treatment decisions and medicolegal considerations.

The purpose of this study was to compare ChatGPT‐4o‐generated and human‐created medical history summaries regarding clinically relevant quality criteria in paediatric orthopaedic practice. These criteria encompass temporal consistency (accurate chronological presentation), spatial consistency (appropriate anatomical localisation), accident description precision, symptom accuracy and specificity, documentation of previous interventions, writing style and overall clinical utility.

It was hypothesised that human‐created history summaries would demonstrate higher quality in critical clinical dimensions such as temporal consistency and accuracy of complex orthopaedic presentations, while AI systems might show comparable performance in stylistic aspects.

## MATERIALS AND METHODS

### Study design and setting

This prospective, randomised, blinded comparative study was conducted at a specialised paediatric orthopaedic practice between January and March 2025. The study protocol was approved by the Ethics Committee of Eastern Switzerland (EKOS 2025‐01475 EKOS 25/122) and conducted in accordance with the Declaration of Helsinki. Written informed consent was obtained from all patients and their legal guardians.

### Sample size calculation

The sample size was determined a priori based on an expected effect size of 0.8 (large effect), alpha = 0.05 and power = 0.80. Using G*Power 3.1 software, a minimum of 15 paired observations was calculated as necessary to detect a statistically significant difference. Twenty patients were included to account for potential data loss and to ensure adequate power.

### Participants

Twenty consecutive patients presenting with knee complaints were included. The mean patient age was 14.2 ± 2.3 years (range 10–17 years), with 11 males (55%) and 9 females (45%). Inclusion criteria were: (1) first‐time consultation at the practice, (2) age between 10‐18 years, (3) knee‐related chief complaint, (4) consent for audio recording and (5) complete medical history available. Exclusion criteria included incomplete audio recordings, technical transcription problems or missing informed consent. All medical histories were conducted by the same experienced paediatric orthopaedic surgeon (C.C.) to minimise variability in history‐taking technique.

### Data collection and processing

#### Audio recording and transcription

All consultation conversations were digitally recorded using secure, HIPAA‐compliant equipment. The average consultation duration was 15 ± 5 min. All conversations were conducted in Swiss German, a distinct German dialect spoken in the German‐speaking regions of Switzerland. Audio recordings were transcribed using MacWhisper Transcription Pro (Good Snooze, Jordi Bruin, Runner Config engine: WhisperKit, model: openai_whisper‐large‐v3, language: German) locally, without an internet connection, to ensure data privacy. All patient‐identifying information was systematically removed during the anonymisation process. Only fully anonymised transcripts were submitted to ChatGPT‐4o (OpenAI) for summary generation.

#### Summary generation

For each patient encounter, two versions of medical history summaries were created, both in standard German (Hochdeutsch) rather than the Swiss German dialect used during the consultations:

##### Human‐generated summaries

The treating orthopaedic surgeon (CC, with >25 years of experience in orthopaedics and creating summaries) created summaries immediately following each consultation using standard clinical dictation practices, without access to the audio transcription. The surgeon followed a similar but not identical structure to the AI prompt, documenting patient information, main complaints, symptom timeline, relevant history, potential triggers, previous treatments and their outcomes.

##### AI‐generated summaries

Anonymised transcripts were processed using ChatGPT‐4o (OpenAI; model version as of January 2025) with the following standardised prompt:

*‘Create a detailed medical history based on the following transcript between a doctor and a patient. The history should include all relevant symptoms experienced by the patient, as well as the temporal sequence of these symptoms. Please consider the following elements:*

1. Begin with general information about the patient, including any pre‐existing conditions.
2. Summarise the patient's main complaints and capture all relevant symptoms.
3. Determine when symptoms first appeared and describe their development over time.
4. Enquire about relevant lifestyle habits of the patient, such as diet, exercise and stress.
5. Examine whether the patient can identify possible triggers or specific situations that influence symptoms.
6. Consider whether the patient has already taken measures to alleviate symptoms, and whether these measures were successful.
7. Ensure all information is captured as precisely as possible to create a comprehensive and accurate history’.

The ChatGPT prompt did not explicitly specify the output language; however, when provided with transcripts in German (automatically converted from Swiss German dialect during transcription), the model consistently generated summaries in standard German.

### Evaluation process

#### Evaluator panel

Three independent orthopaedic specialists with extensive clinical experience served as evaluators. All evaluators received standardised training on the evaluation criteria and scoring methodology.

#### Blinding and randomisation

All 40 summaries (20 human‐generated, 20 AI‐generated) were randomised and anonymised for evaluation. Evaluators were blinded to the source of each summary throughout the assessment process.

#### Evaluation methodology

For each case, evaluators first listened to the original audio recording to establish ground truth, then evaluated both corresponding summaries. The study assessed the accuracy and completeness of information transfer from the original consultation to the written summary.

#### Assessment criteria

Eight quality dimensions were evaluated using a 6‐point Likert scale (1 = very poor, 6 = excellent), which corresponds to the Swiss school grading system. This familiar grading scale was deliberately chosen to enable evaluators to orient themselves within an assessment framework they had known since childhood, potentially improving consistency and intuitive understanding of the rating categories:

1. Temporal consistency: Accurate chronological presentation of symptom onset and progression.
2. Spatial consistency: Appropriate anatomical localisation and spatial relationships.
3. Accident description: Precise documentation of injury mechanisms and circumstances.
4. Symptom accuracy: Factual correctness of reported symptoms and complaints.
5. Symptom specificity: Level of clinical detail and orthopaedic relevance.
6. Previous interventions: Completeness of prior treatment documentation.
7. Writing style: Clarity, organisation and appropriateness for a medical context.
8. Overall impression: Holistic assessment of clinical utility and quality.

### Statistical analysis

Statistical analyses were performed using JMP 18.0 (SAS Institute). Data normality was assessed using the Shapiro–Wilk test. Since data were not normally distributed (*p* < 0.001), paired Wilcoxon signed‐rank tests were used for all comparisons and a Bonferroni correction for multiple comparisons was applied. Interrater reliability was calculated using intraclass correlation coefficients (ICC) with 95% confidence intervals. Effect sizes were calculated using Cohen's *d*, computed as the mean difference divided by the pooled standard deviation. Although Cohen's *d* traditionally assumes normality, it was used here as a standardised measure of effect magnitude to facilitate comparison with other studies; the primary inferential statistics relied on nonparametric tests. Effect sizes were interpreted according to Cohen's criteria (small: 0.2, medium: 0.5, large: 0.8). Measurement accuracy was ± 0.5 points on the 6‐point scale; results are reported to one decimal place consistent with this precision. Statistical significance was set at *α* = 0.05.

## RESULTS

### Overall quality assessment

The analysis encompassed 60 evaluations (3 evaluators × 20 patient cases) for each summary type. Human‐generated summaries received significantly higher overall ratings compared to AI‐generated summaries (5.2 ± 0.8 vs. 4.5 ± 0.8, mean difference 0.7 points, 95% CI: 0.5–0.9, *p* < 0.001). The effect size (Cohen's *d* = 0.8) indicated a large practical difference between the two approaches.

### Criterion‐specific analysis

Human‐generated summaries demonstrated statistically significant superiority in four of eight evaluated criteria after Bonferroni correction for multiple comparisons (Table 1). The largest performance gaps were observed in accident description (1.0‐point difference), spatial consistency (0.9 points) and temporal consistency (0.7 points). No significant differences were found in writing style or in the documentation of previous interventions, with both approaches receiving notably low scores on this criterion.

**Table 1:** Comparison of quality ratings between human‐generated and ChatGPT‐4o‐generated medical history summaries.

Evaluation criterion	Human‐generated mean ± SD (95% CI)	ChatGPT‐generated mean ± SD (95% CI)	Mean difference (95% CI)	*p*‐value	Cohen's *d*
Temporal consistency	5.5 ± 0.6 (5.3–5.6)	4.8 ± 1.5 (4.4–5.1)	0.7 (0.3–1.1)	<0.001	0.48
Spatial consistency	5.2 ± 1.1 (4.9–5.4)	4.3 ± 1.2 (4.0–4.6)	0.9 (0.5–1.3)	<0.001	0.58
Accident description	5.6 ± 0.8 (5.4–5.8)	4.7 ± 1.7 (4.2–5.1)	1.0 (0.5–1.4)	<0.001	0.57
Symptom accuracy	5.1 ± 1.0 (4.8–5.3)	4.7 ± 1.3 (4.3–5.0)	0.4 (0.1–0.8)	0.026*	0.29
Symptom specificity	4.8 ± 1.1 (4.5–5.1)	4.4 ± 1.2 (4.1–4.7)	0.4 (0.0–0.8)	0.035*	0.28
Previous interventions	3.0 ± 1.7 (2.6–3.4)	3.2 ±± 1.9 (2.7–3.6)	−0.2 (−0.7 to 0.4)	0.603	−0.07
Writing style	4.7 ± 1.0 (4.4–4.9)	4.7 ± 0.8 (4.5–5.0)	−0.1 (−0.4 to 0.3)	0.768	−0.04
Overall impression	5.2 ± 0.8 (5.0–5.4)	4.5 ± 0.8 (4.3–4.7)	0.7 (0.5–0.9)	<0.001	0.80

*Note*: *p*‐values calculated using paired Wilcoxon signed‐rank tests. Cohen's *d* is reported as a standardised effect size measure. Abbreviations: CI, confidence interval; SD, standard deviation.

* Not significant after Bonferroni correction for multiple comparisons.

### Inter‐rater reliability

Interrater reliability among the three evaluators was moderate (ICC = 0.64, 95% CI: 0.52–0.74) for overall impression ratings. Individual criteria showed varying reliability, ranging from 0.42 (previous interventions) to 0.71 (temporal consistency). All three raters independently demonstrated directional agreement favouring human‐generated summaries, with mean rating differences of 0.65 (Rater 1), 0.58 (Rater 2) and 0.87 (Rater 3) points on the overall impression scale (Table 2).

**Table 2:** Inter‐rater reliability and evaluator‐specific rating differences.

Rater	Overall rating difference*	95% CI	Significant criteria (*n*/8)	ICC for overall impression
1	0.65 ± 0.18	0.28–1.02	5	0.64 (95% CI: 0.52–0.74)
2	0.58 ± 0.22	0.13–1.03	4	
3	0.87 ± 0.19	0.48–1.26	6	

ICC, intraclass correlation coefficient.

* Human‐generated minus ChatGPT‐generated scores.

### Error pattern analysis

Qualitative analysis of poorly rated ChatGPT summaries was performed by the senior author (C.C.), who remained blinded to the AI/human assignment during this analysis. Temporal inconsistencies (e.g., conflicting timelines for symptom onset) were identified in 14 of 60 evaluations (23%). Omission of relevant accident details occurred in 21 of 60 evaluations (35%). Inaccurate symptom localisation was noted in 11 of 60 evaluations (18%). Hallucination of information not mentioned in the original consultation was detected in 7 of 60 evaluations (12%).

## DISCUSSION

The most important finding of this study was that human‐generated medical history summaries significantly outperformed ChatGPT‐4o in critical clinical dimensions, including temporal consistency (0.7‐point difference), spatial consistency (0.9‐point difference) and accident description accuracy (1.0‐point difference), while showing comparable performance in writing style.

These findings align with recent evaluations of LLMs in clinical settings. Williams et al. [18] reported similar challenges with AI‐generated emergency department summaries, noting overlapping error types including inaccuracies, hallucinations and omissions. Hopkins et al. [5] found that while AI dictation systems achieved low word error rates, they still required human oversight for optimal performance. The observed performance gaps were particularly pronounced in areas requiring contextual interpretation and synthesis of complex orthopaedic presentations, suggesting that human clinicians possess superior capabilities for recognising implicit relationships and integrating scattered clinical information.

An important consideration for future research is the potential role of retrieval‐augmented generation (RAG) technology in improving AI documentation quality. RAG allows LLMs to access curated, domain‐specific knowledge bases rather than relying solely on general training data, potentially reducing hallucinations and improving accuracy [4]. Woo et al. demonstrated that RAG‐enhanced models can achieve accuracy comparable to specialist physicians on orthopaedic questions [12, 19]. Oettl et al. [11] emphasised that small language models with agentic capabilities may offer advantages in orthopaedic applications, while the same authors [10] provided a comprehensive review of generative AI applications and challenges in orthopaedics.

The results have several important implications for orthopaedic surgeons considering AI implementation. The observed deficits in temporal and spatial consistency could have significant clinical consequences in orthopaedic practice, where accurate assessment of injury mechanisms and symptom progression timelines is crucial for diagnosis and treatment planning. Misrepresentation of accident details or symptom localisation could lead to inappropriate diagnostic workups or treatment decisions.

Rather than replacing human documentation, AI systems may be most valuable as drafting tools in hybrid workflows. ChatGPT's comparable performance in writing style suggests potential utility for initial draft generation, with mandatory physician review and editing before finalisation.

Paediatric orthopaedics presents unique documentation challenges, including developmental considerations and complex family dynamics. The ability to capture subtle clinical nuances—such as parental concerns or child behaviour patterns—remains a distinctly human capability that current AI systems cannot replicate.

A critical limitation identified in this study is the loss of nonverbal communication in transcript‐based AI processing. Experienced orthopaedic surgeons routinely observe and incorporate nonverbal cues into their clinical assessments, including pain behaviours demonstrated during consultation, parent‐child interactions providing diagnostic insights, subtle signs of anxiety and gait abnormalities. This clinically significant contextual information is absent from AI‐generated summaries based solely on verbal transcripts. Such limitations suggest that future medical AI systems will require multimodal approaches incorporating audio, video and potentially biometric data to approach human‐level clinical documentation quality [16].

The moderate inter‐rater reliability observed in this study (ICC = 0.64) reflects a fundamental characteristic of human evaluation processes. Despite differences in evaluation stringency among the three assessors, all demonstrated a consistent directional preference for human‐generated summaries. This agreement in tendency, combined with variability in absolute ratings, actually strengthens the robustness of the main findings.

Beyond documentation quality, this study raises critical concerns about AI's potential impact on fundamental orthopaedic reasoning processes. For resident physicians and orthopaedic trainees, there is a particular risk of cognitive degradation from automation [20]. The development of essential orthopaedic competencies—spatial reasoning, biomechanical analysis and pattern recognition—could be compromised if trainees become overly dependent on AI‐generated documentation and interpretations [7, 8, 9].

### Limitations

Several limitations should be acknowledged. The single‐centre design with 20 patients limits generalisability to other orthopaedic practices and patient populations. The moderate inter‐rater reliability (ICC = 0.64) reflects the inherent subjectivity of quality assessment despite standardised evaluation criteria. Despite the blinding protocol, complete masking of summary authorship may have been challenging, as AI‐generated text can exhibit recognisable stylistic patterns, representing a potential source of detection bias. The study focused exclusively on knee complaints in paediatric patients, potentially limiting applicability to other orthopaedic conditions or adult populations. Additionally, the text‐based nature of AI processing inherently excludes nonverbal communication signals that experienced orthopaedic surgeons routinely incorporate into their clinical assessments.

The automatic conversion of Swiss German dialect to Standard German during transcription may have resulted in subtle information loss, thereby affecting the quality of the source material for both summary types. The ChatGPT‐4o model used in this study represents technology from early 2025, and newer models may demonstrate improved performance. The generic prompt employed was not optimised for orthopaedic documentation, and domain‐specific prompt engineering could potentially enhance AI performance.

Finally, this study evaluated documentation quality but did not assess downstream clinical outcomes, cost‐effectiveness or long‐term impacts on physician workflows.

### Clinical relevance

For day‐to‐day clinical work, these findings suggest that AI‐assisted documentation should be implemented through hybrid workflows with mandatory physician oversight rather than autonomous AI documentation. Physicians should specifically verify temporal sequences, spatial descriptions and injury mechanisms during review, as these were the areas with the highest AI error rates. Institutions should implement quality audits and training on recognising AI error patterns [15]. Complex paediatric presentations should continue to rely on traditional physician documentation until AI technology matures.

## CONCLUSION

Human‐generated medical history summaries significantly outperformed ChatGPT‐4o across multiple quality dimensions in paediatric orthopaedic practice. While AI demonstrated acceptable stylistic capabilities, substantial deficits in temporal consistency, spatial accuracy and clinical detail capture indicate that current LLMs are not ready for unsupervised implementation in orthopaedic documentation workflows.

The findings support a cautious approach to AI integration, emphasising hybrid workflows where artificial intelligence assists rather than replaces human clinical judgement. For orthopaedic surgeons considering AI adoption, robust quality assurance mechanisms and mandatory physician oversight remain essential.

Future research should investigate whether RAG systems, multimodal AI approaches incorporating visual and audio data and speciality‐specific fine‐tuning can address the observed limitations. Longitudinal studies examining the impact of AI‐assisted documentation on trainee development and clinical outcomes are warranted.

## AUTHOR CONTRIBUTIONS

**Carlo Camathias:** First draft; supervision; design of study; final draft; **Kata Papp:** Data collection; corrections second draft. **Patrick Betschart:** Data collection; corrections second draft. **Berhard Speth:** Corrections final draft, design of study. **Victor Valderrabano:** Corrections final draft; design of study. **Elias Ammann:** Data collection; corrections final draft.

## CONFLICT OF INTEREST STATEMENT

The authors declare no conflicts of interest.

## ETHICS STATEMENT

Ethikkommission Ostschweiz (EKOS) (2025‐01475 EKOS 25/122). Informed consent was obtained from all individual participants included in the study and their legal guardians.
